# Micro-CT Microcalcification Analysis: A Scoping Review of Current Applications and Future Potential in Breast Cancer Research

**DOI:** 10.3390/tomography10110126

**Published:** 2024-10-24

**Authors:** Redona Brahimetaj, Jan Cornelis, Bart Jansen

**Affiliations:** 1 Department of Electronics and Informatics (ETRO), Vrije Universiteit Brussel (VUB), 1050 Brussels, Belgium; jpcornel@etrovub.be (J.C.); bart.jansen@vub.be (B.J.); 2 IMEC, 3001 Leuven, Belgium

**Keywords:** micro-computed tomography, breast cancer, breast microcalcifications

## Abstract

Micro-computed tomography (micro-CT) is a non-destructive imaging technique that offers highly detailed, 3D visualizations of a target specimen. In the context of breast cancer, micro-CT has emerged as a promising tool for analyzing microcalcifications (MCs), tiny calcium deposits that can indicate at an early stage the presence of cancer. This review aimed to explore the current applications of micro-CT in analyzing breast MCs (ex vivo, animal models, and phantoms) and to identify potential avenues in scientific research. We followed PRISMA guidelines for scoping reviews, yielding 18 studies that met our criteria. The studies varied in their purposes: feasibility and optimization of micro-CT for breast cancer imaging and MC analysis/diagnosis, comparison with other imaging modalities, development of micro-CT scanners and processing algorithms, enhancement of MC detection through contrast agents, etc. In conclusion, micro-CT offers superior image quality and detailed visualization of breast tissue (especially tumor masses and MCs), surpassing traditional methods like mammography and approaching the level of detail of histology. It holds great potential to enhance our understanding of MC characteristics and breast pathologies when used as a supplementary tool. Further research will solidify its role in clinical practice and potentially expand its applications in breast cancer studies.

## 1. Introduction

Micro-computed tomography (micro-CT) is a 3D imaging technique that has found many (new) application opportunities in biomedical research [1] during the last decade. It allows the analysis and visualization of the internal and external structures of a target specimen in great detail and high resolution. The underlying principle of micro-CT is similar to that of a conventional CT. An X-ray beam passes through an object/region of interest (ROI) and a detector captures the photons that are not absorbed and not deflected by scattering to create several 2D projections from multiple different angles. The projection images are then processed using a reconstruction algorithm to produce a 3D model of the specimen. However, there are key differences with conventional CT imaging: the X-ray radiation dose used in micro-CT is significantly higher and exposure times are longer, resulting in spatial resolution that far exceeds that of conventional CT imaging. Over the years, micro-CT technology has advanced significantly, allowing current scanner resolution to range between 0.5 and 50 μm [2]. Micro-CT has been employed in various medical fields [3], including cancer research, due to its ability to provide detailed insight into tissue structure and composition. Specifically, in the context of breast cancer, micro-CT has been used in a few studies aiming to characterize breast microcalcifications (MCs), which are small calcium deposits in breast tissue that serve as early indicators of the disease. The early detection and detailed characterization of MCs are crucial for improving diagnostic accuracy. The precise analysis of their morphology can provide important information about the presence of breast cancer, thereby enabling timely diagnosis and treatment [4]. Currently, breast MCs are typically analyzed using mammography, the standard screening modality for their detection. However, the micro-structure of MCs remains unresolved due to the limited resolution of mammography and the inherent limitations of evaluating these structures in 2D. An imaging modality like micro-CT is particularly valuable in this case, as its high-resolution and 3D imaging capabilities can provide some detailed structural insights that mammography cannot.

Given the critical role of MCs in early breast cancer detection and our research group’s focus on advancing their analysis beyond what conventional modalities allow, we conducted a scoping review of relevant articles to provide an overview of the applications of micro-CT in the analysis of breast MCs. This scoping review provides an overview of the applications of micro-CT in breast MC analysis, highlighting previous achievements and discussing potential future directions.

## 2. Methodology

We conducted a systematic scoping review following the Systematic Reviews and Meta-Analysis extension (PRISMA) [5]. All authors agreed on the study protocol outlined in the following sections.

### 2.1. Search Strategy

We performed a literature search in Scopus, Web of Science, and PubMed up to 30 July 2024. Only English language studies were included. We wrote three queries in total, each one designed to fit the specific syntax requirements of the resource platform. The search query (provided in Appendix A) consists of the following terms: ‘micro-CT’, ‘X-ray microtomography’, ‘microCT’ OR ‘micro-computed tomography’, ‘micro computed tomography’, ‘microcalcifications’, ‘micro-calcifications’, ‘microcalcification’, ‘micro-calcification’, ‘calcifications’, ‘calcification’, ‘MCs’, ‘MC’, and ‘breast’ in combination with the AND/OR Boolean operators. We have optimized our search queries to retrieve only articles containing the relevant keywords in their title, abstract, or keywords, thereby minimizing the need for manual exclusions afterward. References of all matched papers were manually searched to ensure the inclusion of every relevant article in the review.

### 2.2. Eligibility Criteria and Study Selection

The articles retrieved from our queries were reviewed solely by R.B (Redona Brahimetaj). The inclusion criterion applied was: all published journal articles reporting on the use of micro-CT in the analysis of breast MCs, including studies conducted on both human and/or animal models and/or phantoms. The exclusion criteria were: (a) non-English publications; and (b) conference papers. No sample size restrictions were imposed. Considering that the aim of this scoping review is to gain a broad understanding of how micro-CT has been utilized to analyze breast MCs, we opted not to apply strict exclusion criteria (e.g., excluding animal models or studies on synthesized MCs). This approach allows for a more comprehensive overview of the existing research. Rayyan [6] reference management software was used to save the articles, assess them for eligibility, and facilitate the full-text review process.

### 2.3. Data Tabulation and Quality Assessment

The following key details were tabulated: study reference (Ref.), micro-CT scanner used, image resolution, number of specimens and/or number of MCs (if reported), study purposes, and conclusions. Where specific data were not clear/available, ‘N/A’ is written. The quality of the articles was evaluated using the Newcastle–Ottawa scale, which assesses studies across three domains: selection, comparability, and outcome [7]. According to this quality scale, a maximum of nine stars/points can be given to a study, and this score represents the highest quality. The extracted scores were categorized into three quality grades: (i) Good: 3–4 stars in selection, 1–2 in comparability, and 2–3 in outcome; (ii) Fair: 2 stars in selection, 1–2 in comparability, and 2–3 in outcome; (iii) Poor: 0–1 stars in selection, 0 in comparability, or 0–1 in outcome.

## 3. Results

**Figure 1:**
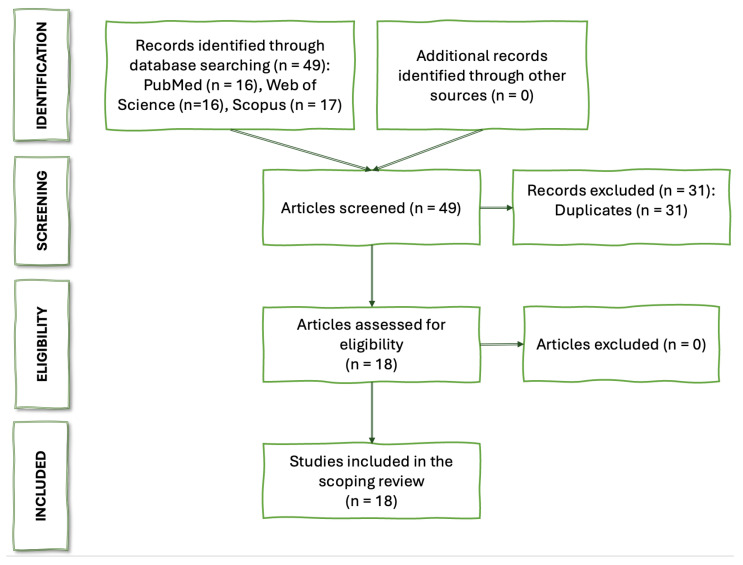
Study flowchart following PRISMA guidelines.

The literature search in Scopus, Web of Science, and PubMed yielded 49 articles in total. Of the 49 abstract titles screened, 31 were excluded as they were duplicates. In total, 18 articles were included in the analysis. The study flowchart is shown in Figure 1.

All of the identified studies used either real MCs extracted from breast tissue or synthesized MCs (calcium carbonate grains) that closely resemble the characteristics of MCs found in real breast tissue. In exploring the diverse applications of micro-CT, the studies covered human tissue, animal models, and synthetic materials. Of the 18 articles, 13 performed ex vivo micro-CT analyses on real MCs extracted from breast tissue [8,9,10,11,12,13,14,15,16,17,18,19,20], 3 analyzed synthesized calcium grains using phantoms [21,22,23], and 2 employed animal models with synthesized MCs [24,25]. The majority of the studies, 11 in total, focused on reporting findings based on MCs clusters [8,9,11,12,13,15,16,19,21,24,25], while the authors of only 7 of the studies performed the analysis of individual MCs in their work [10,14,17,18,20,22,23]. Only two studies reported breast cancer diagnosis (benign/malignant) based solely on the properties of individual breast MCs scanned at high resolution [18,20]. A variety of micro-CT scanner models from different manufacturers were used. The most commonly used model was Skyscanner, while other scanners such as eXplore Locus, Scanco Medical AG (micro-CT80), and Phoenix X-ray vtomex were also used. The spatial resolution used in the studies varies widely, ranging from 4 to 200 μm. The quality assessment score of the included studies (based on the Newcastle–Ottawa quality scale) is provided in Table 1. All 18 studies were assessed with a ‘good’ quality score. The main study characteristics are listed in Table 2. Unless specified, ‘specimen’ refers to human specimens. As shown, while not all studies were primarily focused on directly analyzing MCs, all reported findings are related to MCs and/or utilized micro-CT in their analysis. When the main conclusions of the analyzed papers are not directly focusing on MC analysis, we report only those conclusions related to MCs. The study purposes can be summarized as follows: (a) feasibility of using a micro-CT scanner to analyze structural and anatomical features of breast cancer specimens (with MCs findings); (b) comparison of micro-CT with breast-CT for MC signal profiles; (c) assessment of tumor margins via micro-CT; (d) development of a micro-CT scanner dedicated to breast imaging; (e) modeling of 3D MC clusters and validation with imaging techniques; (f) assessment of resolution impact and diagnosis using MCs scanned with micro-CT; (g) investigation of contrast agents for enhanced MC detection in micro-CT; (h) optimization of micro-CT for detecting calcium grain properties in breast CT; and (i) development of reconstruction algorithms for breast micro-CT imaging.

## 4. Discussion

Micro-CT is an imaging technique that offers high resolution (μm-level) 3D visualizations of small specimens. It is especially useful for analyzing subtle morphological features that conventional imaging cannot detect. In breast cancer research, it is particularly valuable for analyzing breast MCs, which are important for the early detection of malignancy. There is still limited knowledge about MCs [4], hence studying them at a μm resolution is essential to understand their intrinsic properties and links with different breast lesions.

While mammography remains the gold standard for image-based detection of MCs, its low resolution limits it to cluster analysis and does not allow detailed examination of individual MCs. Histological analysis, though the gold (tissue-based) standard for breast cancer diagnosis, is labor-intensive, destructive, and cannot provide a comprehensive volumetric evaluation of biopsy samples [19]. In contrast, micro-CT enables high-resolution, near-histological volumetric imaging, enabling a more detailed examination of MC morphology.

In this scoping review, we evaluated all articles that used a micro-CT scanner to analyze breast MCs, whether as the primary study focus or as an indirect objective, while there exist studies focusing on the current clinical applications of micro-CT in various fields (i.e., cardiovascular imaging and lung carcinoma) [26,27]—to the best of our knowledge—*this is the first scoping review conducted on the applications of micro-CT in analyzing (breast) MCs.* There has been a lack of a review on this specific topic, primarily due to the limited number of studies that have taken this analysis path. Our work addresses this gap, providing a valuable contribution to the field. The review should serve as a resource for researchers in the field of medical imaging, breast oncology, radiology, and biomedical engineering, as well as those developing advanced imaging technologies and CAD systems, while Table 2 presents detailed information, in this section, we provide a narrative overview of the article’s conclusions within the three categories listed below. We highlight the most relevant insights into current knowledge. Additionally, we also focus on the key overarching limitations identified and suggest future directions.

### 4.1. Micro-CT Analysis of Breast MCs in Human Specimens

The micro-CT analysis of MCs in human specimens represents the highest number of articles in our review. We have categorized the numerous conclusions reported into those focused on (a) the usage and/or technical feasibility of micro-CT scanners in analyzing MCs, and (b) those investigating MC characteristics and their diagnostic implications from a micro-CT perspective.

*Usage and Technical Feasibility of Micro-CT Scanners:* The first study that scanned breast MCs with a micro-CT scanner and realized that they can be detected by both visual inspection and via a CAD system at a high level of detail was performed in 2004 [8]. Subsequent studies continued to explore similar and/or other objectives. It has been reported that micro-CT scanners can (i) effectively differentiate breast tissue components at a level of details comparable to histology [9]; (ii) detect more MCs than X-ray projection imaging, which (may) miss some [10]; (iii) create realistic 3D models of MC clusters, which can then be used to simulate MC clusters in 2D mammography and 3D DBT [11]; (iv) visualize tumor masses and MCs in real time [12]; (v) accurately locate MCs on paraffin blocks [15] and assess their distance to the specimen margins [12]; and (vi) be used to perform high-resolution 3D inspection of paraffin blocks, with the potential to aid pathologists in diagnosing breast lesions [19]. Additionally, in [19], it is reported that using a micro-CT scanner with propagation-based phase-contrast techniques can improve MC detection.

*MC Characteristics and their Diagnostic Implications—a Micro-CT Perspective:* Benign and malignant MCs differ internally—benign MCs are trabecular and larger, malignant MCs are smaller and amorphous [10]. The presence of many small MCs may suggest malignancy. Malignant MCs have a more irregular shape than benign ones [14]. Microtexture analysis of breast MCs assessed with a micro-CT scanner has the potential to refine BIRADs classification [16]. Using only the shape feature of (individual) MCs assessed by a micro-CT scanner is not sufficient to distinguish between benign and malignant lesions [17]. Only two studies have specifically exploited the diagnostic potential of only using (radiomic) features extracted from individual MCs to diagnose (employing machine learning algorithms) breast cancer [18,20]. Even though the purposes of these two studies were slightly different, it was concluded that individual breast MCs can be used to diagnose breast cancer patients [18]. Moreover, individual MC texture features are more important than pure shape features for distinguishing between benign and malignant MCs [18]. In [20], the assessment of the impact of (simulated) image resolution to diagnose MCs into benign/malignant demonstrated that higher resolutions significantly improve classification accuracy, where the highest resolution assessed (8 μm) provided the best results.

The studies analyzing MCs in human specimens are highly important as they use data that directly reflect real-world clinical data/conditions, providing essential validation. However, all the reviewed studies are limited by small sample sizes, ranging from 1 to 103 specimens, and a lack of standardized micro-CT scanning protocols for paraffin-embedded samples. Both these limitations may affect the generalizability of their findings. More cautious claims and thorough validation are needed before drawing firm conclusions. For example, the authors of [10] report that benign MCs are larger than malignant ones, yet their reported size exceeds 1 mm, contradicting the definition of MCs (less than 1 mm). Similarly, the authors of [20] emphasize the impact of resolution, but it remains uncertain to what extent the simulated resolution matches actual scanning conditions (the study acknowledges this limitation). Another major limitation is that all the reviewed studies primarily focus on highly suspicious cases identified in mammography screenings. These specimens have already been flagged as likely malignant, hence creating an important bias in the dataset by under-representing purely benign MCs. To complement this, while our review targets micro-CT MC analysis, it is important to note that some breast carcinomas (including DCIS cases), either may not be calcified at all, or MCs are present but too small to be detected by mammography, leading to missed diagnostic information. The absence or under-detection of MCs in mammographies presents a critical diagnostic challenge that is currently not fully addressed. In itself, this fact highlights the need for complementary approaches—at least in research—to quantify in depth how much is missed in mammography but detected with micro-CT. For example, two studies [10,14] demonstrate that micro-CT detects more MCs than mammography, highlighting its potential to uncover overlooked information. Another limitation is that MC clusters, typically located in the breast’s terminal ductal units and considered less prognostically unfavorable than those in larger ducts, are not or are only sporadically analyzed in the reviewed studies, limiting the comprehensiveness of the analysis and missing valuable insights into prognosis. Lastly, although there are two key types of MCs (amorphous and psammoma-body-like), most studies have focused solely on amorphous MCs, potentially overlooking significant prognostic differences.

### 4.2. Micro-CT Analysis of MCs in Animal Models

In 2008, an animal model (Fischer 344 rat) was used to quantify tumor growth and MC deposition over time. BMP-2 growth factor protein was used to produce MCs. It was concluded that (i) BMP-2 is capable of initiating and inducing breast MCs, and (ii) tumor growth is exponential over the 5 weeks of monitoring. With respect to MCs, they were measurable only starting in week 3, growing rapidly, and plateauing by week 4 [24]. In [25], an ex vivo tissue model was developed by injecting varying concentrations (to better mimic breast tissue heterogeneity) of calcium HA into murine mammary glands of FVB female mice. This study demonstrated that (i) BP-Au NP contrast agent can significantly enhance X-ray attenuation; (ii) the X-ray attenuation increased linearly with increasing HA concentration; and (iii) BP-Au NPs have the potential to improve MC detection on mammography.

Both articles present very interesting study purposes. However in [24], the authors do not analyze how the MC depositions’ characteristics evolve over the monitored days. Additionally, both studies use synthesized HA MCs, which raises questions about their resemblance to MCs in human specimens. The growth patterns or detection of benign MCs remain unexplored, as HA is typically associated with malignancy. Additionally, though speculative, it seems unrealistic [28] to assume that MC growth in humans plateaus by week 4, as reported in [24].

### 4.3. Micro-CT Analysis of Synthesized Calcium Grains in Phantoms

In [25], the authors analyzed MCs with varying concentrations of HA using an in vitro imaging phantom. They showed that BP-Au NPs enhanced the contrast for detecting MCs, and hence, they suggested that there is a potential for improved mammographic MC detection using this contrast agent. In 2016, a customized micro-CT scanner (BμCT) was designed. Validation was carried out using a breast phantom (CIRS mod. 1272-00-00, appendix material of [21]). It was demonstrated that MCs and soft lesions are visible even when using a low radiation dose. In [22,23], micro-CT was used to assess the signal profiles of synthesized calcium grains for validating a breast-CT system. It was concluded that the signal profiles from low-noise breast-CT images are similar to those from micro-CT [22]; there is a strong correlation in MC detectability between the micro-CT and breast-CT imaging techniques [22]; signal profiles from micro-CT imaging can be combined with statistical properties to assess the performance of other imaging modalities under consideration [23]; and the detectability of MCs is significantly influenced by the morphological parameters of calcium grains [23].

Performing phantom studies is quite important as they allow for controlled and reproducible setups. However, the studies we reviewed have limitations, such as overlooking the significance of the type of the MCs analyzed within their phantom. For example, in [21], the authors do not report if the MCs they use in their phantom show a more malignant or benign-like appearance. This is important when evaluating new scanners as benign/malignant MCs have different morphologies that vary in their detectibility [23].

### 4.4. Future Directions

Recent studies are using micro-CT to analyze the tumor margins of extracted tissue after surgery [29]. We anticipate that given their proximity to the excised tumor area, these specimens contain MCs. Establishing open-access databases of micro-CT images is crucial to fully exploit tissue/MC morphology. This is especially important because, despite the wealth of research ideas, the high costs of micro-CT scans—whether for purchase or rental—limit their use.

The majority of studies we reviewed focus on MC clusters, with only a few reporting findings related to individual MCs, while the emphasis on clusters is clearly valuable—they are strongly associated with malignancy—it is crucial to also study individual MCs. Understanding their formation is fundamental—clusters originate from individual MCs, and their growth patterns could provide critical insights into even earlier stages of malignancy. Additionally, understanding what drives the formation of MCs remains a significant gap in the field. Although human-based growth analysis cannot be conducted in vivo, animal models could offer valuable insights into these processes [24]. Having temporal/subsequent 3D high-resolution micro-CT images of individual breast MCs would enable the development of computational models to simulate their growth dynamics and analyze how they differ in benign/malignant conditions. These models, particularly if combined with MC chemical composition analysis, could provide valuable insights into the early stages of MC formation and the factors driving their development.

Micro-CT’s resolution far surpasses that of mammography, and it will take many years for mammography to reach this level (if ever). Even if higher resolution in mammography can be achieved, it remains inherently a 2D modality. Although DBT is a 3D imaging modality, its resolution is still limited compared to micro-CT, which provides both 3D imaging and high-resolution. Recent advancements in both dedicated breast-CT and DBT show promise [21,22,30,31], despite technical limitations. Future work in further optimizations (i.e., resolution enhancement, optimization of scanning protocols, radiation dose reduction, improved patient comfort, etc.) is important and should continue.

While mammography remains essential for breast cancer detection, micro-CT could serve as a complementary tool for detailed analysis in complex cases, offering insights beyond what mammography can currently provide. In future work, it would be interesting to quantify the diagnostic improvement if future mammography were to achieve a resolution comparable to micro-CT. Only one study [20] has attempted to assess the impact of image resolution on breast cancer diagnosis using MC properties, concluding that the highest diagnostic accuracy occurs at the highest resolution considered. However, it remains unanswered at what resolution the performance plateaus. A limitation of this study was that three out of the four studied resolutions were simulated rather than obtained from actual scans. Future research should focus on scanning MCs at varying real resolutions to better assess their diagnostic value. Moreover, while various research paths have been explored, deep learning approaches for detecting or classifying MCs in high-resolution 3D micro-CT images remain largely unexplored, presenting a significant opportunity for future studies.

The high-resolution 3D volumetric capabilities of micro-CT can potentially enhance analysis by preserving MCs that might be lost in histological procedures (e.g., due to damage caused by chemical agents or microtome slicing). Micro-CT also provides a comprehensive 3D view, potentially helping pathologists in making more informed decisions and providing guidance tho tissue cutting [19]. Consequently, micro-CT has significant potential to be used as an ex vivo complementary tool in clinical practice and advanced clinical research, offering additional insights regarding MCs, which are currently lacking.

Micro-CT has significant potential as a supplementary tool to conventional modalities, but several practical aspects must be addressed for its successful integration into clinical workflows. First, its role should be seen as complementary to conventional mammography and histology, particularly in cases where conventional imaging is inconclusive or more detailed analysis is required. In terms of safety, for ex vivo use, there are no risks to patients since radiation is applied only to the specimen. However, specialized training will be necessary for radiologists and technicians to operate and interpret micro-CT images effectively, as micro-CT introduces more complexity compared to the traditional imaging that they are used to analyzing. Additionally, micro-CT’s cost-effectiveness must be evaluated to ensure its benefits justify the increased costs and that its use remains warranted as more currently hidden information becomes available.

## 5. Conclusions

This scoping review summarizes the current applications of micro-CT imaging in breast cancer research, with a particular focus on MC characterization and analysis.

Based on the reviewed studies, we conclude that micro-CT offers superior image quality and precise visualization of MCs, surpassing traditional methods like mammography and approaching the level of detail of histology. This indicates a strong potential for future studies on micro-CT to enhance MC analysis beyond the performances of current diagnostic methods and to explore fundamental questions on cancer pathologies that (to date) remain unanswered.

Further research is needed to investigate the role of micro-CT in the workflow of clinical practice and to expand its applications in breast cancer studies.

## Figures and Tables

**Table 1:** Summary of studies analyzed using the Newcastle–Ottawa scale.

Ref.	Selection (4 *)				Comparability (2 *)	Exposure (3 *)			Total (9 *)
	**1**	**2**	**3**	**4**	**5**	**6**	**7**	**8**	
2004, [8]	*		*		**	*	*		6
2008, [24]	*	*	*		**	*	*		7
2011, [9]	*	*	*	*	**	*	*	*	9
2011, [10]	*	*	*	*	**	*	*	*	9
2011, [11]	*	*	*	*	**	*	*		8
2013, [12]	*	*	*	*	**	*	*	*	9
2013, [13]	*	*	*		**	*	*		7
2013, [25]	*		*		**	*	*		6
2014, [14]	*	*	*	*	**	*	*	*	9
2014, [15]	*	*	*		**	*	*		7
2016, [21]	*		*		**	*	*		6
2016, [16]	*	*	*	*	**	*	*	*	9
2017, [17]	*	*	*	*	**	*	*	*	9
2020, [22]	*	*	*		**	*	*		7
2021, [23]	*	*	*		**	*	*		7
2022, [18]	*	*	*	*	**	*	*	*	9
2023, [19]	*	*	*		**	*	*		7
2023, [20]	*	*	*	*	**	*	*	*	9

(1) Specimen diagnoses confirmed by biopsy or standard method; (2) studies analyze >10 MCs; (3) experimental procedures are well-documented; (4) both benign and malignant MCs are included; (5) studies provide qualitative or quantitative micro-CT analysis (*), or both (**); (6) micro-CT procedure is clearly defined; (7) micro-CT is applied uniformly across all diagnoses; and (8) studies consider the most common histological diagnosis.

**Table 2:** Micro-CT applications in breast MC analysis. EBCCD—Electron Bombarded Charged Coupled Device; CAD—Computer-Aided Diagnosis; DCIS—Ductal Carcinoma In Situ; IDC—Invasive Ductal Carcinoma; BMP-2—Humoral Bone Morphogenetic Protein 2; DBT—Digital Breast Tomosynthesis; SCM—Shaved Cavity Margins; BP-Au NPs—bisphosphonate functionalized gold nanoparticles; HA—hydroxyapatite.

Ref.	Scanner	Resolution	Samples	Purpose	Conclusions
2004, [8]	Customized (EBCCD)	N/A	1 mastectomy specimen; MCs nr:N/A	(1) Evaluate micro-CT reconstruction algorithms (cone-, fan-, and parallel-beam) and evaluate them for MC detection.	(1) Cone-beam algorithm offers superior image quality and MC detection. (2) Micro-CT-scanned MCs can be detected by both visual inspection and using a CAD system.
2008, [24]	eXplore Locus	90 μm (whole animal); 45 μm (tumor ROI);	8 female Fisher344 rat; MCs nr:N/A	(1) Assess BMP-2’s role in MC formation; (2) Determine whether it exerts its biological activity systemically or only locally; (3) Develop a robust animal model for the testing of new diagnostic agents for breast cancer.	(1) BMP-2 can act as a humoral factor to produce MCs in breast cancer; (2) It is unclear if BMP-2 mRNA affects MCs via cancer cells, tumor osteoblasts, or surrounding stroma.
2011, [9]	SkyScan 1072	17 μm	15 specimens (7 DCIS, 3 IDC, 5 benign); MCs nr:N/A	(1) Assess micro-CT feasibility for fine breast tissue structure; (2) Correlate with histology.	(1) Micro-CT effectively differentiates breast tissue components (parenchyma, adipose, tumor, MCs) at a micro-structural level, comparable to histology. (2) Significant differences exist between all tissue pairs except fibrous tissue vs. fibroglandular parenchyma.
2011, [10]	SkyScan 1072	17 μm	16 specimens: 11 malignant, 5 benign; MCs nr:N/A	(1) Analyze MC interior structure with micro-CT.	(1)Benign and malignant MCs differ internally: benign MCs are trabecular; malignant MCs are granular/amorphous. (2) Micro-CT detects more MCs than X-ray projection imaging. (3) Benign MCs are larger (0.1–2.7 mm) than malignant MCs (0.05–0.5 mm).
2011, [11]	SkyScan 1172	17–30 μm	23 biopsy specimens: 13 malignant, 10 benign; 54 MCs clusters.	(1) Develop 3D models of various MC cluster types; (2) Validate model realism in patient images using 2D mammography and digital breast tomosynthesis.	(1) Micro-CT creates realistic 3D models of MC clusters. (2) These 3D models can be used to simulate accurate MC clusters in 2D mammography and 3D tomosynthesis.
2013, [12]	SkyScan 1173	N/A	103 breast cancer specimens; MCs nr:N/A	(1) Explore micro-CT usage (in real time) for assessing mass and MC spatial orientation relative to tumor margins in lumpectomy specimens.	Micro-CT accurately visualizes tumor masses/MCs within specimens and relative to tumor margins.
2013, [13]	SkyScan 1173	N/A	25 SCM specimens with lumpectomy; MCs nr:N/A	(1) Determine if micro-CT can accurately identify tumors in lumpectomy SCM.	(1) Micro-CT findings match pathology 92% of the time. (2) Unlike specimen radiography, micro-CT accurately assesses MC distance to the tumor margin.
2013, [25]	Scanco Medical AG, micro-CT80	10 μm; 100 μm	1 in vitro phantom, 1 ex vivo female rat; MCs nr:N/A	(1) Investigate BP-Au NPs for contrast-enhanced radiographic detection of breast MCs.	(1) BP-Au NPs contrast agent provides high contrast for detecting MCs in both in vitro phantom and ex vivo rat model. (2) X-ray attenuation increases linearly with HA calcium concentration.
2014, [14]	Skyscan 1076	35 μm	11 biopsy specimens, MCs nr:597	(1) Evaluate the 3D shape of individual breast MCs using high-resolution micro-CT and compare findings with pathological analysis.	(1) Micro-CT enhances the understanding of malignant and benign MC morphology. (2) Many small MCs may indicate malignancy. (3) Malignant MCs are more irregular in shape than benign ones.
2014, [15]	Nikon XT H225	33 μm	12 biopsy specimens; 31 MCs	(1) Demonstrate/design methods on how to locate MCs within tissue specimen.	(1) Both micro-CT and X-ray fluorescence effectively locate MCs on the surface of paraffin-embedded tissue blocks; (2) Micro-CT offers optimal tissue and marker visibility, depth selectivity, spatial resolution, and scan time.
2016, [21]	Customized	50 μm, 100 μm, 200 μm	1 phantom; MCs nr:N/A	(1) Develop a micro-CT scanner dedicated to the breast.	(1) The developed breast-dedicated micro-CT scanner produced high-resolution images, comparable to existing breast-CT scanners. (2) It detected MCs and soft lesions at a low radiation dose (similar to mammography).
2016, [16]	Phoenix X-ray vtomex	6 μm	31 biopsy specimens (11 DCIS, 20 benign); MCs nr:N/A	(1) Explore the sensitivity of X-ray dark-field contrast for clinical MC assessment.	(1) Dark-field mammography may allow in situ assessment of MC clusters in native (within the body) breast tissue, focusing on morphology rather than chemical composition for absorption and scattering. (2) Microtexture analysis of MCs could refine subjective BIRADS classification, improving cancer risk stratification and reducing unnecessary biopsies.
2017, [17]	SkyScan 1176	9 μm	29 biopsy specimens; MCs no:829	(1) Evaluate if 3D mathematical modeling of MC structure can predict malignancy.	(1) The 3D shape of MCs does not distinguish benign lesions from those with unknown malignant potential or breast cancer. (2) No morphological parameters or MC types showed statistical correlation with B-classification of lesions.
2020, [22]	MicroXCT-200	34 μm	MCs no: 35 MCs (synthetically generated from calcium carbonate grains)	(1) Quantitatively compare signal profiles of MCs acquired using a breast-CT against a micro-CT scanner.	(1) Signal profiles from low-noise breast-CT images are comparable to micro-CT after spatial resolution correction. (2) MC detectability shows high correlation between both micro-CT and breast-CT.
2021, [23]	MicroXCT-200	34 μm	3 phantoms with MCs clusters; MCs no: 58 MCs grains in total (synthetically generated)	(1) Demonstrate micro-CT’s utility for determining MC size/shape to evaluate detection performance in breast-CT.	(1) Signal profiles from low-noise breast-CT images are comparable to those from micro-CT after adjusting for spatial resolution. (2) MC detectability demonstrates a high correlation between micro-CT and breast CT.
2022, [18]	Skyscan 1076	9 μm	94 biopsy specimens; MCs no: 3504;	(1) Analyze the association of individual breast MC shape and texture with malignancy. (2) Evaluate MCs’ potential to diagnose benign/malignant patients.	(1) High-resolution micro-CT analysis reveals a strong link between breast malignancies and individual MCs. (2) MC texture features in transform domains more effectively classify benign/malignant MCs than shape features.
2023, [19]	Propagation-based phase contrast	4 μm	2 entire breast mastectomy specimens (DCIS). MCs no: N/A	(1) Optimize propagation-based phase-contrast CT for multiscale X-ray imaging of the breast.	(1) MCs are clearly visible in propagation-based phase-contrast micro-CT scans. (2) 3D inspection of entire paraffin-embedded blocks offers complementary help to pathologists, aiding in breast lesion diagnosis by overcoming challenges like MC dissolution or tissue damage during processing.
2023, [20]	Skyscan 1076	8 μm	86 biopsy specimens; MCs no: 707-3457	(1) Explore the impact of image resolution (8-original, 16, 32, 64 simulated) on diagnosing breast cancer using radiomic features of individual MCs.	(1) The highest classification results are achieved when analyzing MC properties at the highest resolution (8 microns). (2) Analyzing MC properties at higher resolutions than those used in current digital mammograms could improve breast cancer diagnosis.

## Data Availability

Data sharing is not applicable.

## Abbreviations

Micro-CT	Micro-computed Tomography
MCs	Microcalcifications
PRISMA	Preferred Reporting Items for Systematic Reviews and Meta-Analyses
ROI	Region of Interest
EBCCD	Electron Bombarded Charged Coupled Device
CAD	Computer-Aided Diagnosis
DCIS	Ductal Carcinoma In Situ
IDC	Invasive Ductal Carcinoma
BMP-2	Humoral Bone Morphogenetic Protein 2
DBT	Digital Breast Tomosynthesis
SCM	Shaved Cavity Margins
BP-Au NPs	Bisphosphonate functionalized Gold Nanoparticles
HA	Hydroxyapatite

## Appendix A. Search Queries Used in the Review

For the literature search conducted in the review, the following queries were employed across different databases:

- Scopus: TITLE-ABS (“micro-CT” OR “micro-ct” OR “X-ray microtomography” OR “microCT” OR “micro-computed tomography” OR “micro computed tomography”) AND TITLE-ABS (“microcalcifications” OR “micro-calcifications” OR “microcalcification” OR “micro-calcification” OR “calcifications” OR “calcification” OR “MCs” OR “MC”) AND TITLE-ABS (breast) AND (DOCTYPE (ar))
- Web of Science: (TI=(“micro-CT” OR “micro-ct” OR “X-ray microtomography” OR “microCT” OR “micro-computed tomography” OR “micro computed tomography”) OR AB=(“micro-CT” OR “micro-ct” OR “X-ray microtomography” OR “microCT” OR “micro-computed tomography” OR “micro computed tomography”))
  AND (TI=(“microcalcifications” OR “micro-calcifications” OR “microcalcification” OR “micro-calcification” OR “calcifications” OR “calcification” OR “MCs” OR “MC”) OR AB=(“microcalcifications” OR “micro-calcifications” OR “microcalcification” OR “micro-calcification” OR “calcifications” OR “calcification” OR “MCs” OR “MC”)) AND (TI=(breast) OR AB=(breast))
- PubMed:(“micro-CT”[Title/Abstract] OR “micro-ct”[Title/Abstract] OR “X-ray microtomography”[Title/Abstract] OR “microCT”[Title/Abstract] OR “micro-computed tomography”[Title/Abstract] OR “micro computed tomography”[Title/Abstract]) AND (“microcalcifications”[Title/Abstract] OR “micro-calcifications”[Title/Abstract] OR “microcalcification”[Title/Abstract] OR “micro-calcification”[Title/Abstract] OR “calcifications”[Title/Abstract] OR “calcification”[Title/Abstract] OR “MCs”[Title/Abstract] OR “MC”[Title/Abstract]) AND (breast[Title/Abstract])
